# Cardiovascular Risk During Pregnancy: Scoping Review on the Clinical Implications and Long-Term Consequences

**DOI:** 10.3390/jcm14217516

**Published:** 2025-10-23

**Authors:** Maria Morales-Suarez-Varela, Francisco Guillen-Grima

**Affiliations:** 1 Research Group in Social and Nutritional Epidemiology, Pharmacoepidemiology and Public Health, Department of Preventive Medicine and Public Health, Food Sciences, Toxicology and Forensic Medicine, Faculty of Pharmacy and Food Sciences, Universitat de València, 46100 Burjassot, Spain; 2 Biomedical Research Center in Epidemiology and Public Health Network (CIBERESP), Carlos III Health Institute, 28029 Madrid, Spain; 3 Department of Health Sciences, Public University of Navarra, Av. Barañain, s/n, 31008 Pamplona, Spain; 4 Group of Clinical Epidemiology, Area of Epidemiology and Public Health, Healthcare Research Institute of Navarre (IdiSNA), 31008 Pamplona, Spain; 5 Department of Preventive Medicine, Clinica Universidad de Navarra, Av. Pío XII 36, 31008 Pamplona, Spain

**Keywords:** cardiovascular risk, pregnancy, obstetric complications, cardiovascular risk factors, clinical management

## Abstract

**Background/Objectives:** Cardiovascular risk (CVR) during pregnancy is an increasingly relevant topic due to its clinical significance and impact on maternal and fetal health. Pregnancy involves substantial physiological changes that may result in adverse outcomes such as gestational hypertension, gestational diabetes, obesity, and preeclampsia. These complications not only increase morbidity and mortality during pregnancy and the early postpartum period but also predispose women to long-term cardiovascular disease (CVD). Therefore, accurate assessment of CVR during pregnancy is essential for identifying risk factors and implementing preventive and therapeutic strategies tailored to the unique physiological context of gestation. To examine CVR during pregnancy and its long-term consequences, addressing etiological, diagnostic, and therapeutic aspects to provide an integrative perspective on the relationship between cardiovascular alterations and pregnancy. **Methods**: A scoping review was conducted in accordance with PRISMA-ScR (Preferred Reporting Items for Systematic Reviews and Meta-Analyses extension for Scoping Reviews) guidelines. Literature searches were performed in PubMed, Embase, and Scopus databases using terms related to pregnancy, cardiovascular diseases, and risk factors. The review covered studies published from 2019 to November 2024. A total of 205 articles were initially identified, of which 20 met the inclusion and exclusion criteria and were selected for analysis. **Results**: Pregnancy is considered a natural “cardiovascular stress test” that can unmask or trigger latent CVR factors. Complications such as preeclampsia, gestational diabetes, and intrauterine growth restriction are associated with a higher risk of developing CVD later in life. Genetic factors, such as telomere length, and specific biomarkers have emerged as promising tools for CVR assessment during pregnancy. Personalized management strategies—including regular monitoring and lifestyle modifications—have shown effectiveness in reducing both immediate CVR and the subsequent development of CVD, particularly in high-risk pregnancies. **Conclusions**: Pregnancy represents a unique window of opportunity for the early identification and management of CVR factors. These risk factors and obstetric complications have significant long-term implications, notably increasing the likelihood of future cardiovascular disease. Preventive and therapeutic interventions initiated during pregnancy are, therefore, crucial for improving maternal and neonatal outcomes and for reducing long-term cardiovascular morbidity.

## 1. Introduction

Cardiovascular adaptations and hormonal changes during pregnancy accommodate fetal demands but may unmask or exacerbate latent cardiovascular disorders, leading to hypertensive disorders of pregnancy such as gestational hypertension and preeclampsia [1,2,3]. Cardiovascular disease remains a leading cause of maternal morbidity and mortality worldwide [4,5], with preeclampsia and gestational hypertension disproportionately affecting women with advanced maternal age, obesity, diabetes mellitus, and a family history of cardiovascular disease [6,7,8,9]. These conditions not only increase perinatal complications but also elevate long-term cardiovascular risk (CVR) for both mother and offspring [6,10,11].

Early diagnosis and appropriate blood pressure control are critical to minimizing adverse outcomes [12,13,14], and emerging evidence implicates nitric oxide dysregulation in the pathophysiology of hypertensive disorders in pregnancy [15,16]. Pre-pregnancy obesity further compounds these risks by promoting insulin resistance, systemic inflammation, and endothelial dysfunction, thereby heightening the incidence of gestational diabetes, hypertension, and preeclampsia [6,17,18,19]. Notably, paternal obesity has also been shown to negatively impact fetal development and contribute to pregnancy complications [20].

The global increase in the prevalence of gestational diabetes reflects broader trends in obesity and sedentary lifestyles [21,22,23]. This condition demands early screening, tailored dietary and lifestyle interventions, and, when indicated, pharmacologic management to prevent both short- and long-term cardiometabolic sequelae [24,25,26,27].

International guidelines emphasize the importance of multidisciplinary prenatal care, regular monitoring, and patient education to prevent and effectively manage these complications [3,14,28]. However, the evolving landscape of cardiovascular research, including novel diagnostic biomarkers and updated treatment protocols, underscores the need for a comprehensive and up-to-date review.

This review aims to synthesize and update the current scientific evidence (2019–2024) regarding CVR during pregnancy. It will focus on identifying key risk factors, examining maternal and fetal outcomes, and evaluating modern clinical strategies. The ultimate goal is to support evidence-based decision-making and reduce pregnancy-related cardiovascular morbidity and mortality.

## 2. Materials and Methods

This article is a comprehensive narrative review with systematic elements. Although it does not meet the formal criteria of a systematic or scoping review, it follows a structured and transparent approach to literature identification, selection, and synthesis. The methodology integrates systematic search components to ensure breadth and reproducibility, while maintaining the flexibility of a narrative review to allow critical appraisal and contextual interpretation of findings.

### 2.1. Search Strategy

This scoping review adhered to the guidelines outlined in PRISMA-ScR (Preferred Reporting Items for Systematic Reviews and Meta-Analyses extension for Scoping Reviews) guidelines statement (Table S1: PRISMA-ScR checklist) and has been registered in Open Science Framework (https://osf.io/4xv7k, accessed on 24 July 2025).

The primary information sources used for this work were EMBASE, MEDLINE/PubMed, and SCOPUS. The time frame for the eligible publications was from January 2019 to November 2024. Searches were performed in *titles*, *abstracts*, and *keywords* using a combination of controlled vocabulary (MeSH and Emtree terms) and free-text terms related to *pregnancy*, *cardiovascular diseases*, and *risk factors*.

An advanced search was conducted in the PubMed/MEDLINE database using the terms “Pregnancy” and “Cardiovascular risk” in the title, combined with the following MeSH terms: “Pregnancy,” “Cardiovascular diseases,” and “Risk factors.” The final search query was as follows:

((((pregnancy[Title]) AND (cardiovascular risk[Title])) AND (pregnancy[MeSH Terms])) AND (cardiovascular diseases[MeSH Terms])) AND (risk factors[MeSH Terms])

A temporal filter was applied to restrict the results to publications from 2019 onwards. This search returned 75 results.

For EMBASE, the search strategy involved selecting the term ‘pregnancy’ as a title term, combined with the keyword ‘cardiovascular disease.’ The PICO framework was used to define the population as ‘pregnancy’ and the intervention as ‘cardiovascular risk.’ The final query was:

‘pregnancy’/exp AND ‘cardiovascular risk’/exp AND ‘cardiovascular disease’/exp AND ‘pregnancy’:ti AND ‘cardiovascular disease’:kw

This search resulted in 63 articles.

A similar search strategy was applied in SCOPUS. The terms “cardiovascular risk” and “pregnancy” were used as title terms, and “cardiovascular disease” and “risk factors” were keywords. Filters were applied to include only English-language articles published between 2021 and 2024 and with a document type of “article” (ar). The final query was:

(TITLE (cardiovascular AND risk AND during AND pregnancy) AND KEY (cardiovascular AND disease AND risk AND factors)) AND PUBYEAR > 2020 AND PUBYEAR < 2026 AND (LIMIT-TO (LANGUAGE, “English”)) AND (LIMIT-TO (DOCTYPE, “ar”))

This search yielded 67 articles. A total of 205 articles were retrieved from the three databases. In the revised analysis, the search strategy was broadened to include relevant terms appearing not only in the title but also in the abstract and keywords. This approach ensured a more comprehensive capture of studies addressing cardiovascular risk during pregnancy and reduced the potential for selection bias related to restrictive title-only searches.

The screening process was performed in three sequential phases: (1) title screening, (2) abstract review, and (3) full-text evaluation. Each record was assessed for relevance according to the predefined inclusion and exclusion criteria, and duplicates were removed. Discrepancies in study eligibility were resolved by consensus among the authors.

### 2.2. Article Selection

The initial step involved removing duplicate records. Reference Manager automatically detected and removed 47 duplicates. The remaining records underwent a two-stage screening process—first by title and abstract, then by full text—conducted independently by two reviewers. Discrepancies were resolved by consensus.

#### 2.2.1. Inclusion Criteria

- Topical Relevance: Articles were included based on a review of their title and abstract, provided their primary focus was CVR in pregnant women, ensuring their relevance to the objective of this review.
- Study Design: Priority was given to original research studies (e.g., cohort studies), as well as systematic and narrative reviews that addressed the topic from clinical, epidemiological, or preventive medicine perspectives.
- Target Population: Studies focusing exclusively on pregnant women were included. Studies involving mixed populations (e.g., men or non-pregnant women) were included only if results for pregnant women were reported separately.
- Language: Only articles published in English or Spanish were selected to ensure complete comprehension and rigorous analysis.
- Publication Date: Articles published between 2019 and 2024 were included to ensure the current relevance of the studies.

#### 2.2.2. Exclusion Criteria

- Lack of Thematic Relevance: Articles that, although mentioning CVR and pregnancy, focused on unrelated topics such as oncology, autoimmune diseases, or other conditions outside of pregnancy were excluded.
- Limited Population Representativeness: Studies with small sample sizes (under 60 participants) or those focused on particular geographic regions or ethnic groups were excluded if their results were not generalizable to the broader context of CVR in pregnant women. The exclusion of studies with fewer than 60 participants was based on the aim to include research with adequate statistical power and generalizability. This threshold was chosen to minimize random variation and enhance the reliability of the reported associations between pregnancy-related factors and cardiovascular outcomes.
- Low Methodological Quality or Inadequate Design: Editorials, letters to the editor, conference abstracts, and case reports were excluded, as were articles lacking structural integrity or presenting incomplete data.
- After applying these criteria, the final selection included n = 20 articles for inclusion in this review.

All retrieved records were screened independently by two authors based on relevance, and discrepancies were resolved by consensus. No formal risk-of-bias scoring was performed, but study quality and evidence strength were qualitatively considered in the synthesis.

### 2.3. Methodological Approach for Literature Analysis

A structured methodology was employed to classify and synthesize the most salient aspects of the included studies, ensuring a thorough and systematic analysis of the selected literature. This approach facilitated the subsequent development of summary tables, enhancing the clarity, interpretability, and comparability of the findings. Each included article was reviewed for: Author and year, Study design and population, Gestational period analyzed, Main objectives and variables, and Key findings and reported limitations.

For each eligible study, additional variables were recorded, including population characteristics, sample size, study period, geographical region, and methodological features such as diagnostic criteria and outcome measurement protocols. This information facilitated a structured qualitative synthesis of findings across studies.

A core thematic framework was established, comprising four principal categories that reflect critical domains of CVR in pregnancy:

1. General Considerations of CVR during Pregnancy. This category encompasses publications that address foundational concepts related to CVR during gestation, including epidemiological data and the conceptual underpinnings of CVR in pregnant populations.
2. Pregnancy Complications and CVR. This group includes studies examining pregnancy-specific complications, such as preeclampsia and gestational diabetes mellitus, and their association with both immediate and long-term cardiovascular outcomes.
3. Genetics and Molecular Biology of CVR Factors in Pregnancy. This thematic area encompasses investigations into the genetic, molecular, and pathophysiological mechanisms underlying CVR during pregnancy, providing a biomedical perspective to the broader clinical understanding.
4. Clinical Management of CVR during Pregnancy. This section comprises literature addressing strategies for preventing, diagnosing, and managing CVR in pregnant individuals, with an emphasis on evidence-based guidelines and implications for obstetric care.

Within each thematic group, studies were further categorized by publication type, distinguishing between original research articles (e.g., cohort or case–control studies) and review articles (including narrative reviews, systematic reviews, and meta-analyses). This dual-level classification supports methodological consistency and contextual interpretation, facilitating the integration of evidence across diverse study designs.

### 2.4. Construction of Summary Tables: Original Research Articles

Standardized tables were developed for original studies to summarize and compare key characteristics across articles. Each table included the following components:

- Author and Year of Publication: For citation and identification purposes.
- Study Design: Type of research conducted (e.g., cohort, cross-sectional, case–control).
- Inclusion and Exclusion Criteria: The Criteria used to define the study population are relevant for assessing external validity.
- Gestational Period Analyzed: Specific trimester(s) or pregnancy stage(s) addressed.
- Study Objective and Primary Variables: The main research aim and the variables employed to evaluate it.
- Principal Findings: Primary results, emphasizing outcomes related to CVR.
- Limitations: The authors reported methodological limitations or identified them during critical appraisal, which aids in assessing study quality and internal validity.

### 2.5. Construction of Summary Tables: Review Articles

For review articles, a separate table format was designed to align with the structure and objectives of this publication type. The table included:

- Author and Year of Publication, Study Design, Gestational Period, Primary Objective, Key Findings, and Limitations: As in the original studies, these fields ensure consistent reporting and comparability.
- Main Focus: Summary of the central topic or hypothesis addressed in the review.
- Number of Included Studies and Time Frame Covered: Total articles reviewed and the temporal scope of the literature search.
- Quality of Evidence: An assessment of the methodological rigor of included studies and the review process, based on criteria such as the Newcastle-Ottawa Scale or other validated quality appraisal tools.
- Secondary Findings: Additional observations or findings beyond the primary objective of the review.

**Figure 1:**
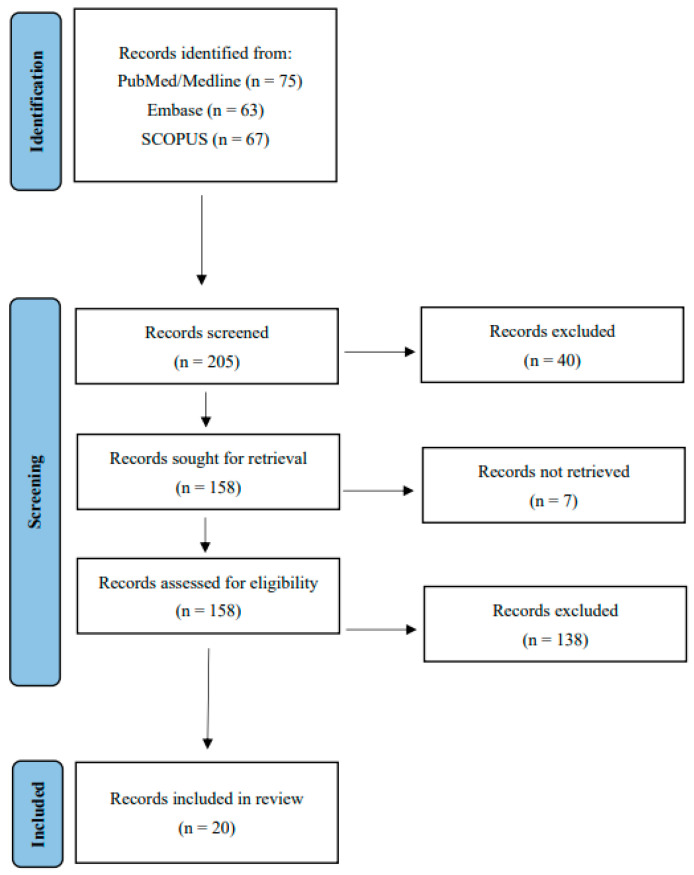
PRISMA flow chart for search and selection of included studies.

At the end of the selection process, a total of 20 studies were included in the review. The complete process of identification, screening, eligibility assessment, and inclusion is summarized in the PRISMA flow diagram (Figure 1).

## 3. Results

The results have been organized into four thematic sections to facilitate the analysis of the impact of CVR during pregnancy. These sections address the general aspects of CVR during pregnancy, including pregnancy complications and their short- and long-term effects, advances in genetics and molecular biology, and CVR biomarkers in pregnant women, as well as clinical management strategies. Below, we detail the most relevant findings from each area, integrating evidence from original studies and reviews. The results are presented in table form, followed by an integrated summary of the evidence obtained from each.

Table 1 and Table 2 show research and review articles, respectively, that examined the general aspects of CVR factors during pregnancy from various perspectives. It can be concluded that pregnancy is considered a stage where the demands on the cardiovascular system (CVS) are increased, making it a period in which conditions that elevate CVR both in the short and long term may emerge or be revealed.

Four research articles regarding methodology were included [29,30,31,32]. One of them [30] has a significantly larger sample size compared to the other three, whose study populations consist of only a few hundred participants, which may limit the generalizability of the results to larger populations.

Most of these studies do not focus on a specific period of pregnancy but examine pregnancy as a whole, with some also covering the early postpartum period. Only the study by Harville et al. [29] also investigates the pre-pregnancy state. Therefore, while these studies may offer a limited view of pregnancy individually, they collectively provide a more integrative temporal perspective.

In terms of results, it is evident that CVR observed during pregnancy largely reflects the pre-existing risk state [29], underscoring the importance of proper prevention and control of CVR factors both during and before pregnancy. Parameters showing the strongest correlation between pre-pregnancy and pregnancy levels include BMI, systolic blood pressure (SBP), diastolic blood pressure (DBP), LDL and HDL cholesterol, and triglycerides. In contrast, the correlation is weaker for glucose and insulin [29].

This finding aligns with the fact that CVRFs such as hypertension, obesity, gestational diabetes (GDM), and elevated CRP levels negatively affect the CVS in pregnant women, influencing cardiac remodeling through increased concentric cardiac hypertrophy and reducing the ability to reverse cardiovascular adaptations that occur during pregnancy [32]. Moreover, these factors, such as preeclampsia (PE) and GDM, also increase the risk of adverse cardiovascular events, such as major adverse cardiovascular events (MACE), which are of particular interest due to their high mortality and predominant occurrence in the immediate postpartum period [30].

Conversely, the ST2/IL33 receptor is associated with better postpartum recovery, suggesting it could be a biomarker for reversing cardiac hypertrophy [32].

It has also been observed that women with higher CVR are more likely to experience preterm birth and ICU admissions [31]. However, for a woman with good control of cardiovascular diseases, different modes of delivery do not impact perinatal mortality, further emphasizing the importance of appropriate monitoring and control in women with CVRFs and cardiovascular diseases during pregnancy [31].

Thus, pregnancy is globally projected as a period of special vulnerability for the CVS, highlighting the importance of pre-pregnancy risk profiles and the short- and long-term consequences of presenting CVRFs and complications during pregnancy.

The only review article found analyzes the impact of parity on CVR [33]. It is a meta-analysis that incorporates data from multiple studies involving over 3 million participants, placing it among the highest levels of evidence in the research pyramid. Specifically, the study by Li et al. [33] demonstrates that a higher number of pregnancies and, therefore, gestations lead to an increased risk of cardiovascular events (CVE), with each childbirth increasing the risk by 4%. Overall, women with children have a 14% higher risk of developing cardiovascular disease (CVD) compared to nulliparous women. These findings suggest that pregnancy is a biological state with a direct impact on long-term cardiovascular health, manifesting as an increase in CVR and CVD.

Moreover, the relationship between the number of births and the relative risk of CVD is not linear but follows a J-shaped curve [33].

Table 3 and Table 4 show original and review publications that examine the association between pregnancy complications and the long-term risk of developing CVD.

Regarding methodology, the study by Honigberg et al. [34] is a cohort study with a large sample size, strengthening the validity of the results. However, all data are derived from the UK Biobank, which may limit the applicability of the evidence to populations from other regions.

The study does not examine CVR during pregnancy itself. Instead, it focuses on the postpartum study of women who had experienced hypertensive disorders of pregnancy (HDP), such as preeclampsia, and whether they have a higher risk of developing CVD later in life. The results show that women with a history of HDPs have a higher risk of a CVD diagnosis. Specifically, this population shows an increased prevalence of coronary artery disease, heart failure, and valvular dysfunction. Thus, it can be concluded that HDPs not only represent a risk during pregnancy but also have long-term cardiovascular consequences for women who have experienced them, highlighting the need for rigorous and prolonged postpartum follow-up.

However, the main limitation of this study is that the inclusion of women with a history of HDP was based on patient testimony rather than clinical records, which may introduce recall bias that limits the evidence. Therefore, further research is needed to study the long-term consequences of pregnancy-related cardiovascular disorders using more detailed clinical records, including comprehensive histories of HDPs.

Regarding methodological analysis, seven narrative reviews are included [35,36,37,38,39,40,41], encompassing over 580 references from the late 20th century to 2024, which provide a comprehensive view of pregnancy complications over recent decades. The reviews found examine how different pregnancy complications, such as HDP, GDM, preterm birth, and low birth weight, are associated with short- and long-term maternal CVR, with some complications, such as myocardial infarction (MI) or cardiogenic shock (CS), being potentially fatal both for the mother and fetus in the immediate term. However, as narrative reviews, they lack clear protocols for article selection and review, which may introduce selection bias, making the evidence less robust.

Although the reviews address different stages of pregnancy, collectively they provide a comprehensive overview of pregnancy, from its onset to the late postpartum period. However, they do not focus on the pre-pregnancy CVR condition, except for the study by Fürniss & Stiller [40], which focuses on pregnant women with congenital heart diseases.

Four studies address the impact of more prevalent cardiovascular complications during pregnancy [35,36,37,39], while three focus on less prevalent but more fatal complications such as myocardial infarction (MI) [41], cardiogenic shock [38], and arrhythmias [40].

In general, all adverse pregnancy events are predisposed to increased CVR. However, more specifically, preeclampsia is associated with a 2–4 times higher risk of developing CVDs such as hypertension and coronary artery disease throughout life [35,36,37,39]. GDM, on the other hand, increases the risk of type 2 diabetes (T2DM) up to 7 times, playing a key role in the increase in CVR [36]. Furthermore, even in women who regain normoglycemia after delivery, there remains an elevated risk for developing CVD [39]. Additionally, mothers who give birth before 37 weeks have a 1.79-fold higher risk of vascular mortality [39].

All the evidence presented in the various studies is consistent. Clearly, it shows that any cardiovascular event or disruption of pregnancy progression has long-term adverse effects on the cardiovascular system. The pathophysiological mechanisms underlying these findings are primarily linked to chronic pro-inflammatory states and endothelial dysfunction [37,39].

In contrast, breastfeeding is proposed as a valuable tool for CVS protection, as it is associated with a significant reduction in CVR in the mother [35,39]. Thus, the importance of promoting breastfeeding as part of postpartum management is emphasized as a strategy to reduce maternal CVR.

Focusing on more specific cardiovascular disorders, the importance of CS is highlighted since, although it is infrequent, it has high maternal and fetal morbidity and mortality. Understanding and considering its primary etiologies, such as maternal cardiomyopathy and arrhythmogenic right ventricular dysplasia, is crucial as these occur primarily during the late stages of pregnancy and peripartum [38].

Regarding MI, it is essential to understand the role of spontaneous coronary artery dissection (SCAD) as the primary etiological factor in young pregnant women [38,41]. The incidence of MI is three to five times higher in pregnant women compared to non-pregnant women of the same age. Moreover, it is associated with a high recurrence rate of cardiovascular events postpartum, as well as complications such as preterm birth. These findings underscore pregnancy as a period of heightened cardiovascular demand, during which abnormal cardiovascular function can result in fatal maternal and fetal outcomes [41].

As for arrhythmias during pregnancy, it is noted that women with congenital heart disease are particularly susceptible to this event, making it the most frequent complication in this population, and strict CV monitoring is recommended [40].

Table 5 and Table 6 present the results from original and review works focused on the genetics, molecular biology, and biomarkers of CV risk factors in pregnant women. The findings from both original studies highlight a significant association between CVR in pregnancy and underlying biological markers or predispositions.

In the prospective observational study by Abu-Awwad et al. [42], pregnant women with CVR factors demonstrated significantly shorter telomere lengths compared to those without such risk factors (mean: 0.3537 vs. 0.5728 megabases; *p* = 0.0458). Furthermore, 32% of fetuses in the high-risk group exhibited intrauterine growth restriction (IUGR). These results suggest that telomere length may serve as a potential biomarker for identifying CVR during pregnancy. However, the study was limited by its small sample size, single-center recruitment, and lack of control over external variables such as lifestyle and stress.

**Table 5:** Genetics, molecular biology, and biomarkers of cardiovascular risk factors in pregnant women—Research articles.

Reference and Author	Methodology	Objectives and Main Variables	Primary Outcome	Secondary Outcomes	Limitations
**[43] Abu-Awwad et al. (2023)**	Prospective observational study conducted between 1 January 2020, and 13 December 2022, published in 2023. Sample: 68 pregnant women aged 18–40 years, divided into: Group 1: 38 women without cardiovascular or vascular disease. Group 2: 30 women with cardiovascular risk or established disease. Inclusion: Pregnant women in the 2nd or third trimester with ≥1 cardiovascular risk factor, undergoing elective cesarean delivery. Exclusion: History of substance abuse, psychiatric disorders, thromboembolic or infectious diseases, participation in other studies within the past 3 months, or use of telomere-shortening drugs. Gestational period: second and third trimesters.	To evaluate whether telomere length is reduced in pregnant women with cardiovascular risk factors (CVRFs) compared to those without. Main variable: Telomere length measured via quantitative polymerase chain reaction (qPCR).	Women with CVRFs exhibited significantly shorter telomere lengths (mean: 0.3537 megabases) compared to those without CVRFs (mean: 0.5728 megabases). Statistically significant difference (*p* = 0.0458).	In the CVRF group, 32% of fetuses presented with intrauterine growth restriction (IUGR). Telomere length may serve as a biomarker of cardiovascular risk in pregnant women. Shorter telomeres are associated with increased morbidity and mortality, including various cancers, cognitive decline, depression, and infections.	Small sample size and potential selection bias due to recruitment from a single hospital. Other factors influencing telomere length, such as diet, lifestyle, and stress, were not controlled.
**[44] Tschiderer et al. (2024)**	Prospective Mendelian randomization study based on cohort data. Utilized UK Biobank data recruited from 2006 to 2010; analysis conducted in 2023. Sample: 221,155 pregnant women, 41,506 nulliparous women, and 223,025 men as negative controls. Inclusion: Women with available genetic data and pregnancy history. Exclusion: Incomplete data or no pregnancy history. Focus on long-term post-pregnancy cardiovascular outcomes.	To analyze the association between genetic predisposition to hypertensive pregnancy disorders (HPDs) and cardiovascular risk (CVR). Main variables: Myocardial infarction (MI), stroke, systolic/diastolic blood pressure (SBP/DBP), and age at hypertension diagnosis.	A genome-wide association study identified links between genetic predisposition to preeclampsia/eclampsia and a 20% increased CVD risk. Gestational hypertension is associated with a 24% increased CVD risk. Genetic predisposition is also linked to increased BP and earlier hypertension diagnosis.	Comparison with nulliparous women and men suggests that risk is not solely attributable to pregnancy, indicating other underlying biological mechanisms. Elevated cholesterol, triglycerides, and metabolic markers are also associated with genetic predisposition to HPDs.	Potential genetic bias due to predominantly European ancestry in the UK Biobank sample. Lack of control over other genetic or environmental confounders.

**Table 6:** Genetics, molecular biology, and biomarkers of cardiovascular risk factors in pregnant women—Review articles.

Reference and Author	Design	Main Focus	Number of Articles and Time Frame	Quality of Evidence	Gestational Period	Main Objective	Key Findings	Secondary Outcomes	Limitations
**[45] Aldo et al. (2024)**	Narrative review published in Clinical Chemistry and Laboratory Medicine (CCLM)	Analysis of the role of specific cardiac biomarkers such as natriuretic peptides and troponins in cardiovascular risk evolution during both normal and complicated pregnancies.	102 articles spanning from 1981 to 2023	Findings are based on observational studies, previous reviews, and small-scale clinical trials. The article highlights methodological heterogeneity, lack of standardization, and small sample sizes, which limit the generalizability of the results.	All pregnancy stages: first, second, third trimesters, as well as peri- and postpartum.	To determine whether the measurement of specific cardiac biomarkers can improve cardiovascular risk assessment in pregnant women, aiming to identify early cardiovascular complications.	NT-proBNP and BNP: levels increase during the first trimester, decrease in the second and third trimesters, and rise again during delivery. Values > 200 ng/L of NT-proBNP are associated with heart failure and hypertensive disorders in women with pre-existing heart conditions. High-sensitivity cardiac troponins remain unchanged in uncomplicated pregnancies but increase in conditions like hypertensive disorders and other cardiovascular complications. Troponins are proposed as risk assessment tools, though further validation is needed.	Measurement of natriuretic peptides and cardiac troponins, when combined with other tests such as ECG, may assist in cardiovascular risk stratification in pregnancy, though with limited utility. Routine screening for these biomarkers is not recommended in early pregnancy for women with hypertension or other cardiovascular diseases.	Heterogeneity in biomarker measurement methods and studied populations. A limited number of studies have evaluated the combined use of biomarkers in pregnant women. Lack of consensus on pregnancy-specific reference values for cardiac biomarkers. There is a need for further research to substantiate the evidence linking biomarkers to cardiovascular risk in pregnancy, as current evidence is weak.

Complementing these findings, the Mendelian randomization study by Tschiderer et al. [43] utilized genetic data from over 221,000 pregnant women in the UK Biobank to examine long-term cardiovascular outcomes. The study found that genetic predisposition to hypertensive pregnancy disorders (HPDs)—including preeclampsia and gestational hypertension—was associated with a 20–24% increased risk of CVD later in life. This genetic susceptibility was also linked to elevated blood pressure, earlier hypertension diagnosis, and adverse metabolic profiles, such as increased cholesterol and triglycerides. The inclusion of nulliparous women and men as controls further supported the conclusion that these risks extend beyond pregnancy itself, pointing to shared genetic and metabolic pathways. Nonetheless, the study’s findings may be limited by its reliance on a predominantly European population and potential residual confounding.

Together, these studies underscore both molecular (telomere shortening) and genetic (HPD predisposition) indicators of CVR in pregnant populations, suggesting the potential for future risk stratification and early intervention strategies, while also emphasizing the need for further research with broader and more diverse populations.

The narrative review by Aldo et al. [45] synthesized findings from 102 articles published between 1981 and 2023, focusing on the use of cardiac biomarkers in pregnancy for CVR assessment. The authors reported that NT-proBNP and BNP levels typically increase during the first trimester, decrease in the second and third trimesters, and rise again around delivery. Values exceeding 200 ng/L of NT-proBNP were associated with an elevated risk of heart failure and hypertensive disorders, particularly in women with pre-existing cardiac conditions.

In contrast, high-sensitivity cardiac troponin levels remained stable in uncomplicated pregnancies but were found to increase in the presence of hypertensive disorders and other cardiovascular complications, suggesting their potential utility in risk stratification. However, the review emphasized that troponins require further validation before being used routinely in pregnancy.

The original and review publications included in Table 7 and Table 8, respectively, examine the management of CV risk in pregnant women. The prospective cohort study by Marschner et al. [46] assessed the effectiveness of a postpartum CVR management program among 156 women with a history of pregnancy complications, including HDP, GDM, and IUGR. Over a six-month follow-up period, 80.5% of women enrolled in the program achieved blood pressure targets (<140/90 mmHg, or <130/80 mmHg for those with type 2 diabetes), compared to 69.2% in the non-participating group.

Participants in the program also demonstrated significant reductions in LDL and total cholesterol, as well as improvements in body mass index (BMI) and waist circumference, indicating enhanced cardiovascular health. Additionally, the intervention group reported healthier lifestyle habits, including increased consumption of fish and olive oil, as well as decreased intake of fast food.

Despite these promising outcomes, the study’s non-randomized design limits causal interpretation. Moreover, selection bias may be present due to the high educational level of participants, and the results may not be generalizable beyond the single Australian healthcare setting in which the study was conducted.

The review studies [46,47,48] agree on identifying pregnancy as a physiological “cardiovascular stress test,” capable of triggering or exacerbating underlying conditions, some of which are critical, such as CS or MI. These complications not only represent immediate risks but also have long-term repercussions by increasing the risk of CVD. Therefore, a multidisciplinary approach to prevention, diagnosis, and therapy is necessary to manage cardiovascular consequences effectively.

## 4. Discussion

The evidence from the various studies analyzed demonstrates that pregnancy is a biological period during which the CVS undergoes significant demands, leading to functional changes and physiological adaptations that pose a unique challenge to cardiovascular function. These dynamics have been consistently reflected throughout the present review [29,30,31,32,33] (Table 1 and Table 2). The discussion section not only provides a critical interpretation of the findings but also examines their clinical and methodological implications, identifies areas for improvement, and offers a comprehensive and applied perspective on the preceding analysis.

The translation of these immediate pregnancy complications into long-term cardiovascular disease (CVD) is driven by persistent mechanistic pathways that initiate or accelerate vascular and cardiac remodeling. Specifically, hypertensive disorders of pregnancy (HDP), such as preeclampsia (PE), and gestational diabetes mellitus (GDM) are strongly linked to chronic underlying processes, including endothelial dysfunction, systemic inflammation, and oxidative stress. Endothelial dysfunction, often resulting from placental maladaptation and an imbalance of angiogenic factors—such as increased soluble fms-like tyrosine kinase-1 (sFlt-1) and decreased placental growth factor (PlGF)—induces sustained alterations in the maternal vasculature. This state, initially a physiological adaptation to pregnancy, leads to reduced nitric oxide (NO) bioavailability, increased arterial stiffness, and microvascular injury, which collectively predispose women to long-term hypertension, coronary artery disease, and stroke.

Furthermore, pregnancy complications are now regarded as an accelerated model of metabolic and cardiovascular aging. GDM, for instance, triggers chronic low-grade inflammation and insulin resistance that persist postpartum, substantially increasing the risk of type 2 diabetes mellitus (T2DM) and subsequent CVD. Similarly, PE is associated with sustained activation of inflammatory and immune pathways, contributing to adverse cardiac remodeling characterized by concentric hypertrophy and impaired diastolic function, thereby reducing the capacity to reverse cardiovascular adaptations established during gestation.

In addition, emerging molecular and genetic evidence supports the involvement of angiogenic and anti-angiogenic factors, inflammatory mediators, and epigenetic regulators—such as specific microRNAs and telomere shortening—in the pathophysiological continuum linking pregnancy complications to long-term cardiovascular remodeling. These biomarkers and molecular pathways may represent promising targets for early identification of women at increased cardiovascular risk and for the development of preventive or therapeutic interventions tailored to this population.

Pregnancy involves a series of physiological changes in the cardiovascular system, including an increase in blood volume, a decrease in vascular resistance, and a 50% increase in cardiac output [8,9,10,11,12]. These adaptations are crucial for meeting maternal-fetal demands during this biological stage. However, these changes also represent a “cardiovascular stress test” [29,30,31,32,33] (Table 1 and Table 2).

Recent studies suggest that both genetic and environmental factors may contribute to inadequate cardiovascular adaptations [42,43] (Table 5). These changes subject the cardiovascular system to stress and overstrain, resulting in increased CVR, which is manifested through various conditions, such as PE and GDM [35,36,37,38,39,40,41] (Table 4).

These conditions themselves constitute an elevated CVR during pregnancy, triggering harmful consequences for the cardiovascular system, which may have immediate fatal effects, such as MI, thromboembolism, or cardiogenic shock [35,36,37,38,39,40,41] (Table 4).

### 4.1. Specific Acute Cardiovascular Complications During Pregnancy

Among these acute cardiovascular events, Takotsubo syndrome (also known as stress-induced cardiomyopathy) deserves particular attention. Although relatively rare, its incidence during pregnancy and the postpartum period has been increasingly reported, often triggered by physical or emotional stress. The syndrome mimics acute myocardial infarction, presenting with chest pain, ST-segment elevation, and elevated troponins, but without obstructive coronary artery disease. Pathophysiologically, it is characterized by transient left ventricular dysfunction—typically apical ballooning—likely resulting from catecholamine-mediated myocardial stunning and autonomic nervous system imbalance [42].

During pregnancy, Takotsubo syndrome may be precipitated by obstetric complications, cesarean delivery, or postpartum hemorrhage, and is frequently associated with preeclampsia, eclampsia, or peripartum cardiomyopathy. Although reversible in most cases, it can lead to serious complications such as cardiogenic shock, arrhythmias, or even death if not promptly recognized.

Awareness and early identification of Takotsubo syndrome are crucial in differential diagnosis, especially when evaluating pregnant or postpartum women presenting with acute chest pain and abnormal ECG or troponin elevations. Echocardiography and cardiac MRI are key diagnostic tools, and supportive therapy focusing on hemodynamic stabilization remains the mainstay of treatment.

The inclusion of Takotsubo syndrome in the spectrum of pregnancy-associated cardiovascular complications broadens the understanding of the multifactorial nature of cardiogenic shock and myocardial injury in this setting, emphasizing the need for multidisciplinary monitoring and management of these patients.

However, pregnancy not only triggers short-term CVR but also has long-term repercussions, as numerous studies show an increased prevalence of various cardiovascular conditions, such as ischemic heart disease, valvular heart disease, vasculopathies, or cardiomyopathies, later in life among women who have been pregnant, compared to those who have not [29,30,31,32,33,34,35,36,37,38,39,40,41] (Table 1, Table 2, Table 3 and Table 4). There is a direct and robust correlation between parity and CVR, such that each pregnancy increases the long-term risk of developing cardiovascular disease (CVD) by up to 4%, clearly illustrating the impact pregnancy has on the cardiovascular system [33] (Table 2).

The impact of these complications extends beyond maternal health, also affecting fetal health and well-being. The risk of intrauterine growth restriction (IUGR), preterm birth, and, in some cases, fetal mortality is increased [35,36,37,38,39,40,41,46,47,48,49] (Table 4, Table 7 and Table 8). These outcomes represent a major global health issue affecting a particularly vulnerable population—pregnant women. This situation underscores the importance of advancing research in this area. Furthermore, the existing evidence suggests the existence of molecular and genetic mechanisms that could be targeted for intervention, should a more comprehensive understanding be attained to control, delay, or even prevent the development and progression of cardiovascular disease [43,44,45,47,48,49] (Table 5, Table 6 and Table 8). Potential biomarkers, such as the ST2/IL-33 receptor and lysyl oxidase, related to cardiovascular remodeling [29,30,31,32] (Table 1), as well as troponins and telomere length, which are associated with cardiovascular remodeling [43,44,45] (Table 5 and Table 6), have been proposed.

On the other hand, it is essential to understand all the available options for the comprehensive management of cardiovascular complications related to pregnancy. It has been demonstrated that measures such as medication, cardiovascular marker monitoring (including blood pressure, glucose levels, and body weight), lifestyle changes, proper nutrition, and the administration of safe medications during pregnancy can result in a significant improvement in the development and prognosis of cardiovascular disease. Women who undergo prevention, treatment, and follow-up programs during pregnancy and postpartum experience significant improvements in their cardiovascular profile and a reduced risk of cardiovascular events and related complications [29,30,31,32] (Table 1). Preconception care is also emphasized, as the correlation between pre-existing CVR and that observed during pregnancy underscores the need to optimize the management of CVR factors before conception [29,30,31,32] (Table 1).

Regarding methodology, the reviewed research articles exhibit significant heterogeneity in sample size, ranging from 68 patients in the case of Abu-Awwad et al. [43], which is a small sample size that may limit statistical power, to hundreds of thousands of participants in studies based on databases such as the UK Biobank [34,44], which strengthens the generalizability of their results.

In terms of study design, observational studies—both retrospective and prospective—predominate, which are extremely useful for identifying associations but, in some cases, are insufficient to establish causality. This fact highlights the need for clinical trials in this area. However, pregnant women are routinely excluded from medical research due to various ethical, legal, and safety considerations. As a result, most of the recommendations related to the management of CVR during pregnancy, as well as the majority of associations identified in this area, arise from observational studies, retrospective analyses, or extrapolations from data obtained from the general population, which significantly limits the development of robust evidence to guide specific and safe interventions.

Regarding clinical practice, the findings of this review have direct implications for the primary and secondary prevention of cardiovascular events associated with pregnancy. Available tools—such as biomarker measurement (troponins, ST2/IL-33, telomere length) or the identification of obstetric histories, including preeclampsia or gestational diabetes—enable the early detection of elevated CVR and the initiation of timely interventions to prevent adverse outcomes. These goals can be achieved through the implementation of personalized management strategies. Additionally, the importance of proper postpartum follow-up for women with CVR factors is emphasized. Cost-effective and straightforward assessments, such as blood pressure monitoring and metabolic profiling, are easy to perform, require minimal time during consultations, and are low-cost. These tools can detect the early onset of cardiovascular complications, thereby preventing diagnostic delays and enabling the development of personalized management plans. Such strategies can reduce or prevent the onset of severe conditions, including myocardial infarction and stroke, which carry a high risk of morbidity and mortality. The value of this lies not only in improving maternal health but also in the unique periods of postpartum and infancy, during which children require considerable care and attention in all vital areas for proper development. Reducing both maternal mortality and morbidity enables these needs to be adequately met. Invalidating sequelae, such as those resulting from a stroke or, in extreme cases, maternal death, can have devastating consequences for the life and development of offspring.

Moreover, most of the studies included in this review were conducted in high-income countries, leaving populations in developing and low-resource settings largely overlooked. The prevalence and expression of cardiovascular complications may differ in these populations due to socioeconomic factors, access to healthcare, and genetic diversity. The lack of data from diverse racial and ethnic groups represents a critical limitation that affects the external validity and generalizability of current evidence. For example, major databases such as the UK Biobank—frequently used in high-impact studies like those by Honigberg et al. and Tschiderer et al.—are predominantly composed of individuals of European descent, which restricts the applicability of their findings to other populations. Certain racial and ethnic groups may also present a higher baseline prevalence of CVR factors such as hypertension or diabetes, and differences in the presentation or severity of conditions like preeclampsia. These gaps highlight the urgent need to expand research to underrepresented populations to develop evidence-based and globally equitable clinical guidelines for managing CVR and CVD during pregnancy.

Regarding areas for improvement and research proposals, there is solid and robust evidence of the direct and bidirectional relationship between pregnancy and CVR. Pregnancy itself represents an increase in cardiovascular demand that may lead to CVD, while conditions that increase CVR complicate pregnancy and long-term maternal health.

However, this evidence is not yet conclusive, and further research is required in fields such as CVR-related biomarkers, gene expression associated with CVR, nutritional supplements during pregnancy, and novel forms of early CVR diagnosis. These studies should follow appropriate and well-reasoned methodologies, prioritizing high-quality evidence such as meta-analyses or clinical trials. In such cases, studies should be designed ethically to include pregnant women, thereby generating robust evidence that applies to this population. Greater knowledge in these fields could open new therapeutic avenues, such as epigenetic interventions or using these biomarkers for early diagnosis.

Moreover, studies should be expanded to include underrepresented populations, as most reviewed studies are conducted in developed countries, leaving populations in developing countries largely overlooked. The prevalence of cardiovascular complications may differ in these populations due to socioeconomic factors, access to healthcare, and genetic differences. Therefore, expanding research into these lower-resource populations is crucial for obtaining a more comprehensive and less biased view of the problem.

### 4.2. Critical Appraisal and Future Research Directions

Looking ahead, research should prioritize the design of high-quality, longitudinal clinical trials that actively include pregnant populations, as they remain significantly underrepresented in cardiovascular studies. There is also an urgent need to establish pregnancy-specific biomarker thresholds to enhance the precision of CVR assessment within the unique physiological context of gestation. Furthermore, future investigations should focus on developing and evaluating interventions tailored to distinct maternal risk profiles, integrating genetic, metabolic, and clinical data to advance personalized preventive strategies. Strengthening multidisciplinary collaboration and ensuring equitable inclusion across diverse populations will be essential to improve the translational relevance and global applicability of future findings.

In summary, pregnancy represents a period of high cardiovascular demand that can initiate or unmask underlying pathologies. However, it also provides a unique opportunity for identifying and managing risk factors, with beneficial effects on maternal and fetal health both in the short and long term. However, to achieve this goal, it is essential to overcome methodological barriers and expand the frontiers of knowledge through more innovative, in-depth, and collaborative research, ensuring optimal and safe care for this particularly vulnerable cardiovascular population.

To enhance the clinical applicability of this review, a summary of evidence-based management strategies for cardiovascular risk during pregnancy is presented below. This table provides practical guidance for clinicians, including preventive, diagnostic, and therapeutic considerations tailored to maternal risk profiles and the different stages of pregnancy and postpartum care (Table 9).

## 5. Conclusions

Cardiovascular risk during pregnancy represents a critical and often underrecognized determinant of long-term maternal health. This review highlights the main risk factors, pathophysiological pathways, and potential preventive and therapeutic strategies that may help reduce future cardiovascular disease in women with pregnancy-related complications.

Although the number of available studies remains limited and heterogeneous in design, the cumulative evidence consistently supports an association between pregnancy complications—particularly preeclampsia and gestational diabetes mellitus—and increased long-term cardiovascular risk. These findings emphasize the importance of early risk assessment, structured follow-up, and multidisciplinary collaboration to improve maternal outcomes.

Future research should focus on longitudinal, multiethnic, and adequately powered studies to confirm these associations and identify modifiable determinants of cardiovascular risk. Expanding data collection across diverse populations and healthcare settings will be essential to strengthening external validity and guiding evidence-based clinical recommendations.

Overall, integrating cardiovascular risk assessment into maternal healthcare frameworks can foster earlier prevention strategies that benefit both women and future generations.

## Figures and Tables

**Table 1:** Overview of cardiovascular risk during pregnancy—Research articles.

Reference Number and Author	Methodology	Main Objective and Variables	Primary Outcome	Secondary Outcomes	Limitations
**[29] Harville et al. (2021)**	Prospective observational cohort study Data collection likely occurred several years before 2021; cohorts began in 2009. Sample: 296 women from the International Childhood Cardiovascular Cohort Consortium. Mean age: 27 years. Inclusion: Women with at least one pre-pregnancy and one pregnancy visit with available data. Exclusion: Missing pre-pregnancy data or incomplete pregnancy information. Pregnancy period: Focus on the first and second trimesters.	To determine whether pregnancy reveals latent cardiovascular damage or independently initiates it. Variables: BMI, SBP, DBP, LDL/HDL cholesterol, triglycerides, glucose, insulin (pre-pregnancy vs. during pregnancy).	Most risk parameters showed correlation (r = 0.29–0.54) between pre-pregnancy and pregnancy measurements, suggesting cardiovascular risk during pregnancy reflects pre-pregnancy levels.	Glucose and insulin showed lower correlations. Stronger correlations were observed in the first and second trimesters compared to the third trimester.	Relatively small sample size; variable time between pre-pregnancy and pregnancy measures; lack of data on health status during pregnancy.
**[30] Beyer et al. (2020)**	Observational cohort study (2010–2015). Sample: 18,151,897 pregnant/postpartum women in the U.S.; 993 diagnosed with pregnancy-associated arterial dissection (PAAD). Data from the U.S. National Readmission Database. Inclusion: Women ≥12 years hospitalized during pregnancy, delivery, or within 42 days postpartum. Exclusion: Records missing discharge date or from out-of-state hospitals. Pregnancy period: Focus on postpartum.	To assess risk factors, timing, anatomical distribution, and outcomes of PAAD. Primary variable: Incidence of PAAD. Secondary: Age, chronic hypertension, tobacco use, GDM, hormone therapy.	Incidence: 5.5 per 100,000. Most common: coronary dissections. 61.5% occurred within 30 days postpartum. Higher risk among women with PAAD: age 32.8 vs. 28; chronic HTN 19% vs. 3.1%; tobacco 13.5% vs. 7.8%, etc.	Incidence increased from 4.46 (2010) to 6.2 (2015). In-hospital mortality: 3.7% with dissections (*p* < 0.001). Highest mortality in aortic dissection (8.6%), followed by coronary (4.2%).	Based on ICD-9-coded administrative data, there is a potential for misclassifying cases. Limited to early postpartum (42 days), lacking long-term follow-up.
**[31] Essa et al. (2023)**	Retrospective cohort study (Jan 2017–May 2022). Sample: 108 pregnant women with known cardiovascular disease (CVD); low risk (n = 41), moderate/high risk (n = 67). Mean age: 32.1 years. Inclusion: Pregnant women >18 years with CVD and at least one prenatal/postpartum echocardiogram. Exclusion: Incomplete data or echocardiograms outside the perinatal period. Pregnancy period: First trimester to 12 weeks postpartum.	To assess delivery mode and maternal CVD risk. Primary variable: Delivery mode. Secondary: Adverse perinatal outcomes.	No significant difference in delivery mode by CVD risk group. Among well-compensated high-risk women, delivery mode did not affect severe maternal morbidity (SMM).	Higher incidence of SMM, ICU admissions, and earlier gestational age at delivery in the moderate/high-risk group. BMI was significantly higher pre-pregnancy in the high-risk group.	Small sample size; single academic center; limits generalizability. Possible underpowering to detect differences.
**[32] Ferreira et al. (2023)**	Prospective cohort study (February 2019–July 2022). Sample: 130 pregnant women (54 with CV risk factors: HTN, GDM, obesity; 76 healthy). Inclusion: Pregnant women >18 years with/without CV risk factors. Exclusion: Multiple gestations, severe chronic diseases, or pregnancy losses. Pregnancy period: First to third trimester, plus follow-ups at 1, 6, and 12 months postpartum.	To assess CV remodeling and its postpartum reversal in women with CV risk factors and identify predictive biomarkers. Main variables: LV mass (LVM), diastolic function (E/e’), arterial stiffness (pulse wave velocity), and biomarkers: CRP, ST2/IL-33 receptor, lysyl oxidase.	LVM and ventricular filling pressures increased during pregnancy and normalized postpartum. HTN and obesity predicted less postpartum LVM reversal; obesity was associated with concentric remodeling.	GDM is linked to long-term arterial stiffness. Elevated ST2/IL-33 correlated with better recovery. CRP in the third trimester is linked to poor LVM reversal. Lysyl oxidase levels rose in the third trimester and at the 6-month follow-up.	The study had a small sample size, with many participants enrolled in the third trimester. The COVID-19 pandemic led to follow-up losses.

**Table 2:** Overview of cardiovascular risk during pregnancy—Review articles.

Ref. No. & Author	Design	Main Focus	Number of Studies & Coverage Period	Evidence Quality	Pregnancy Period Considered	Main Objective	Key Findings	Secondary Findings	Limitations
**[33] Li et al. (2019)**	Meta-analysis of cohort studies	Quantitative assessment of the association between parity and cardiovascular risk	10 cohort studies; total of 3,089,929 participants and 150,512 cardiovascular disease (CVD) cases. Included studies published from 1978 to 1 June 2018	Newcastle–Ottawa Scale scores ranged from 7 to 9, indicating high-quality evidence	Not focused on a specific gestational period; parity is considered as a cumulative reproductive history	To determine the relationship between parity and long-term cardiovascular risk, including dose–response analysis	Parity was associated with a 14% increased risk of CVD compared to nulliparous women (RR: 1.14). Each additional birth was associated with a 4% increase in CVD risk. A J-shaped relationship was observed between the number of births and CVD risk.	Similar associations were found for increased risk of ischemic heart disease and stroke. Subgroup analyses revealed variability in risk depending on geographic location and CVD subtype.	Significant heterogeneity in the included studies regarding parity comparisons and dose–response analysis. Not all studies adjusted for potential confounders such as lifestyle, ethnicity, or age at first birth.

**Table 3:** Pregnancy complications and their association with short- and long-term cardiovascular risk—Research articles.

Reference No. & Author	Methodology	Objective & Key Variables	Primary Outcome	Secondary Outcomes	Limitations
**[34] Honigberg et al. (2019)**	Prospective observational cohort study based on the UK Biobank cohort. Data were collected between 2006 and 2010; the study was published in December 2019, with an average follow-up period of 7 years post-data collection.	Population: 220,024 women aged 40–69 years with ≥1 live birth; 2808 (1.3%) had a history of hypertensive disorders of pregnancy (HDP). Inclusion Criteria: Women with and without HDP. Exclusion Criteria: Women with congenital heart disease. Pregnancy Period: Postpartum; focused on histories of gestational hypertension, preeclampsia, eclampsia, and HELLP syndrome.	To determine the relationship between a history of HDP and the development of cardiovascular disease (CVD), compared to women without HDP. Primary Variable: Incidence of CVD, including coronary artery disease (CAD), heart failure, aortic stenosis, and mitral regurgitation. HR: 1.3 (95% CI: 1.04–1.60; *p* = 0.02).	HDP is associated with increased risk of: - CAD (HR: 1.8) - Heart failure (HR: 1.7) - Aortic stenosis (HR: 2.9) - Mitral regurgitation (HR: 5.0) No significant associations with atrial fibrillation, peripheral artery disease, or venous thromboembolism.	- Self-reported history of HDP may introduce recall bias. - Selection bias due to the UK Biobank cohort. - Lack of data to stratify different types of HDP.

**Table 4:** Pregnancy complications and their relationship with short- and long-term cardiovascular risk—Reviews.

Author (Year)	Design	Main Focus	No. of Articles/Period Covered	Quality of Evidence	Pregnancy Period	Main Objective	Key Findings	Secondary Findings	Limitations
**[35] Brown & Smith (2020)**	Narrative review	Association between pregnancy complications and future CV risk	51 articles (2000–2019)	Epidemiological studies, meta-analyses, and large cohorts	Entire pregnancy	Identify pregnancy complications increasing CV risk; emphasize postpartum care.	The history of preeclampsia, GDM, and preterm/low birth weight is linked to higher future CV morbidity and mortality.	Breastfeeding is linked to long-term CV protection; CV risk monitoring should begin postpartum.	Non-systematic review; dependent on selected studies’ quality and heterogeneity
**[36] Graves et al. (2019)**	Narrative clinical review	Pregnancy complications as early CV risk indicators	54 articles (2001–2018)	Clinical guidelines, systematic reviews, and cohort studies	Pregnancy and early postpartum	Identify CV risk after HDP and suggest strategies in primary care	HDP increases HTN risk ×4 and CVD ×2; GDM increases T2DM risk ×7	Follow-up at 6 and 12 months postpartum, including BP, lipids, lifestyle counseling, and awareness posters proposed	No formal quantitative analysis or meta-analysis; generalizability limited
**[37] Staff et al. (2024)**	Narrative review	HDP and maternal CV risk	88 articles (1976–2024)	Varied; includes population-based and systematic reviews	Entire pregnancy and immediate postpartum	Assess HDP and future CV risk; propose follow-up strategies	Women with HDP have 2–4× higher long-term CVD risk	Increased risk of T2DM, kidney disease, and offspring CV risk; use of angiogenic biomarkers and lifestyle strategies recommended	Article selection method not detailed; studies focus on high-income countries
**[38] Greer et al. (2024)**	Narrative review	Cardiogenic shock in pregnancy	80 articles (2005–2023)	Observational records, national registries, prior reviews	Pregnancy and delivery	Guide the identification and management of cardiogenic shock	PPCM, AFE, SCAD as major causes; associated with maternal and neonatal morbidity and preterm birth	SCAI classification adapted for pregnancy; multidisciplinary care essential (e.g., ECMO, IABP)	Lacks a systematic review protocol; evidence is mainly observational
**[39] Quesada et al. (2023)**	Narrative review	Adverse pregnancy outcomes (APOs) and CV risk	101 articles (2005–2022)	Includes robust meta-analysis (13+ million women), plus small, lower-quality studies	Pregnancy through puerperium	Identify APOs as CV risk factors; recommend prevention strategies	Preeclampsia: +63% lifetime CVD risk; GDM: ×2 CVD risk; preterm birth: ×1.79 CV mortality	APOs double the risk of stroke, HF, and CV mortality; breastfeeding reduces HTN, T2DM, and future CVD	Unclear definition of APO; study heterogeneity; mechanisms insufficiently detailed
**[40] Fürniss & Stiller (2021)**	Narrative review	Arrhythmia risk in congenital heart disease (CHD) during pregnancy	34 references (1995–2019)	Multicenter registries, observational studies	Entire pregnancy and up to 6 months postpartum	Identify arrhythmia risk and guide management in CHD	Arrhythmias in 7–9% of CHD pregnancies; >30% in complex CHD; mostly supraventricular	Preconception CV evaluation and Holter monitoring advised; restrict antiarrhythmic drugs in the first trimester	Focus on moderate/severe CHD; lack of data on mild CHD; limited evidence on pharmacologic management
**[41] Gédéon et al. (2022)**	Narrative review	Myocardial infarction (MI) in pregnancy	166 articles (2005–2022)	Observational studies, meta-analyses, registries	Third trimester and immediate postpartum	Update on MI in pregnancy, including risk, diagnosis, and management	Pregnancy increases MI risk 3–5× vs. nonpregnant peers; SCAD is the most common cause; cardiac troponins are effective	Vaginal delivery preferred unless instability; conservative management in SCAD; standard STEMI/NSTEMI treatment applied	MI is rare; most studies are small and heterogeneous; RCT data are lacking

**Table 7:** Management of cardiovascular risk in pregnant women—Research articles.

Reference and Author	Methodology	Objectives and Main Variables	Primary Outcome	Secondary Outcomes	Limitations
**[46] Marschner et al. (2023)**	Prospective cohort study conducted between May 2021 and October 2022. Sample: 156 women aged 30 to 55 years (mean age: 41 years) with a history of pregnancy complications. Inclusion: Women diagnosed with hypertensive disorders of pregnancy (HDP), gestational diabetes mellitus (GDM), or intrauterine growth restriction (IUGR) between January 2013 and December 2020. Exclusion: Women with known cardiovascular disease, unstable medical conditions, cognitive impairment, recent breastfeeding, or planning pregnancy during the study. Postpartum follow-up of women with prior pregnancy complications.	To determine whether a postpartum follow-up and management program improves the cardiovascular profile of women with a history of pregnancy-related cardiovascular complications. Main variables: Blood pressure (BP) and total cholesterol/HDL-cholesterol ratio.	After 6 months, 80.5% of women enrolled in the cardiovascular risk management program achieved BP targets (<140/90 mmHg or <130/80 mmHg for those with type 2 diabetes), compared to 69.2% of women who did not follow the program. LDL and total cholesterol levels were reduced, alongside improvements in BMI and waist circumference. Findings support the utility of women-focused cardiovascular health programs for effective risk factor control and CVD prevention.	Participants in the follow-up program also showed improved lifestyle habits, including increased consumption of fish and olive oil, and reduced intake of fast food.	The study was non-randomized, which may introduce bias and limit its causal inference. Potential selection bias due to high educational levels among participants. The intervention was conducted by the Women’s Heart Clinic in Australia so that results may be specific to that regional healthcare setting.

**Table 8:** Management of cardiovascular risk in pregnant women—Reviews.

Reference No. and Author	Design	Main Focus	No. of Articles and Time Frame	Evidence Quality	Gestational Period	Main Objective	Key Findings	Secondary Findings	Limitations
**[47] Man et al. (2023)**	Narrative review published in Pflügers Archiv—European Journal of Physiology.	Effect of dietary supplements on vascular function in pregnant women and hypertensive disorders of pregnancy (HDP).	Over 250 articles from 1980 to 2023.	Includes meta-analyses providing robust evidence for calcium and omega-3 supplementation effects, supported by clinical trials and observational studies. Animal models were included, albeit with limited clinical applicability.	Entire pregnancy, with emphasis on early to mid-gestation and early postpartum.	To investigate how dietary supplements improve vascular function and cardiovascular risk in pregnant women, focusing on oxidative stress, endothelial dysfunction, and angiogenic factors.	L-arginine and L-citrulline enhance NO availability, lowering BP and improving vascular function. Omega-3 reduces inflammation and oxidative stress, improving placental angiogenesis. Calcium supplementation reduces preeclampsia incidence by 32%. Resveratrol shows promise but needs further validation. Some supplements exhibit epigenetic effects.	Preeclampsia is linked to imbalanced angiogenic factors and oxidative stress, leading to vascular/placental dysfunction. Supplements like vitamins D, C, and E reduce inflammation, though results are inconsistent. Animal studies support L-citrulline to prevent hypertension and placental dysfunction.	High heterogeneity among studies; causality of nutritional deficiencies unclear; limited human data; key findings from animal studies.
**[48] Lumsden (2022)**	Narrative review.	Overview of main CV risk factors during pregnancy and strategies for management.	94 references from 1982 to 2021.	Many recommendations are based on international guidelines, offering strong evidence. Some are from retrospective studies with weaker support.	Entire pregnancy, from preconception to immediate postpartum, focusing on the second and third trimesters.	Provide guidance for managing CV risk factors in pregnant women through early identification and trimester-specific interventions.	Hypertension: differentiated management with safe medications (methyldopa, labetalol). Diabetes/obesity: strict glycemic control and weight/nutrition management. Dyslipidemia: lifestyle changes; statins limited. Thromboembolism: prophylaxis in at-risk women.	CV risk management prevents severe conditions (eclampsia, preterm birth) and improves long-term CV health.	Lack of data for subpopulations (e.g., racial differences); no clear selection/review methodology for included studies.
**[49] Shah et al. (2023)**	Narrative review published in Current Cardiology Reports.	Approach to common CV complications during pregnancy and their management.	133 articles from 2000 to 2023.	Strong evidence for hypertension and preeclampsia based on AHA guidelines. Limited evidence for hyperlipidemia and MI management.	All pregnancy stages, including preconception and postpartum.	Provide comprehensive guidance for diagnosis, treatment, and prevention of pregnancy-related CV complications.	Chronic HTN: increases risk for preeclampsia and preterm birth. Managed with BP control and low-dose aspirin. HDP: requires intensive monitoring and antihypertensive therapy. Severe cases need magnesium sulfate. Hyperlipidemia: monitor lipids preconceptionally. MI: rare but serious; similar initial treatment as in non-pregnant women. PE: managed with LMWH.	Multidisciplinary cardio-obstetric teams recommended. Lifestyle counseling reduces maternal morbidity.	Limited studies on some topics; need for more clinical trials; some data derived from guidelines, others from observational studies.

**Table 9:** Practical clinical recommendations for cardiovascular risk management during pregnancy.

Clinical Aspect	Recommendations/Practical Considerations
Risk assessment	Conduct preconception or early pregnancy screening for hypertension, diabetes, obesity, dyslipidaemia, and a history of preeclampsia or cardiovascular disease. Use validated pregnancy-specific CVR tools where available.
Lifestyle interventions	Promote balanced nutrition, adequate physical activity (as tolerated), weight optimization, smoking cessation, and management of psychosocial stress.
Monitoring	Regular blood pressure monitoring, glucose tolerance testing, and serial assessment of weight gain and proteinuria in high-risk women
Pharmacological management	Select antihypertensive and antidiabetic therapies with established safety in pregnancy (e.g., labetalol, methyldopa, insulin). Avoid contraindicated drugs such as ACE inhibitors, ARBs, and statins.
Delivery and postpartum care	Plan delivery in a facility equipped for maternal cardiac monitoring. Continue follow-up postpartum to monitor resolution or persistence of cardiovascular changes.
Long-term prevention	Encourage cardiovascular follow-up after pregnancy complications (e.g., preeclampsia, gestational diabetes) to reduce future CVD risk through early lifestyle or pharmacological interventions.

## Data Availability

No new data were created.

## Supplementary Materials

The following supporting information can be downloaded at: https://www.mdpi.com/article/10.3390/jcm14217516/s1, Table S1: PRISMA-ScR checklist.

## Abbreviations

The following abbreviations are used in this manuscript:

AFE	Amniotic fluid embolism
APO	Adverse pregnancy outcome
BMI	Body mass index
BP	Blood pressure
CAD	Coronary artery disease
CCLM	Clinical Chemistry and Laboratory Medicine
CHD	Congenital heart disease
CS	Cardiogenic shock
CVD	Cardiovascular disease
CVR	Cardiovascular risk
CVRF	Cardiovascular risk factor
CVS	Cardiovascular system
ECG	Electrocardiogram
ECMO	Extracorporeal membrane oxygenation
GDM	Gestational diabetes mellitus
HDP	Hypertensive disorders of pregnancy
HR	Hazard ratio
HTN	Hypertension
IABP	Intra-aortic balloon pump
ICD	International Classification of Diseases
ICU	Intensive care unit
IUGR	Intrauterine growth restriction
LDL	Low-density lipoprotein
LMWH	Low molecular weight heparin
LVM	Left ventricular mass
MACE	Major adverse cardiovascular events
MI	Myocardial infarction
NSTEMI	Non–ST-segment elevation myocardial infarction
NT-proBNP	N-terminal pro-B-type natriuretic peptide
OSF	Open Science Framework
PAAD	Pregnancy-associated arterial dissection
PE	Preeclampsia
PPCM	Peripartum cardiomyopathy
PRISMA	Preferred Reporting Items for Systematic Reviews and Meta-Analyses
qPCR	Quantitative polymerase chain reaction
RR	Relative risk
SCAI	Society for Cardiovascular Angiography and Interventions
SCAD	Spontaneous coronary artery dissection
SBP	Systolic blood pressure
SMM	Severe maternal morbidity
STEMI	ST-segment elevation myocardial infarction
ST2/IL-33	Suppression of tumorigenicity 2/Interleukin-33 receptor
T2DM	Type 2 diabetes mellitus
