# Supplementary material for: Cardiovascular Risk During Pregnancy: Scoping Review on the Clinical Implications and Long-Term Consequences
Source: J Clin Med. 2025 Oct 23;14(21):7516. doi: 10.3390/jcm14217516 (PMC12609239; doi:10.3390/jcm14217516)
Supplement: Supplementary file 1 [file jcm-14-07516-s001.zip › jcm-3936166-supplementary.pdf]

Supplementary Material

PRISMA-ScR checklist

"Cardiovascular Risk During Pregnancy: Scoping Review on the Clinical Implications and Long-Term Consequences."

Item	PRISMA-ScR Requirement	How It Was Addressed in the Manuscript	Section / Page
**1. Title**	Identify the report as a scoping review.	The title specifies "Scoping Review."	Title
**2. Abstract**	Provide a structured summary including background, objectives, methods, results, and conclusions.	The abstract is structured and includes all required elements.	Abstract
**3. Introduction – Rationale**	Describe the rationale for the review in the context of what is already known.	Explains the clinical importance of cardiovascular risk (CVR) during pregnancy and the need for an updated synthesis.	Introduction
**4. Objectives**	State explicit review questions and objectives.	"To examine CVR during pregnancy and its long-term consequences..."	Abstract / Introduction
**5. Eligibility Criteria**	Specify characteristics of sources (e.g., years, language, study design).	Inclusion/exclusion criteria are clearly stated: 2019–2024, English/Spanish, studies on pregnant women with CVR.	Methods 2.1.1–2.1.2
**6. Information Sources**	Describe all information sources and the date of last search.	Searches conducted in PubMed, Embase, and Scopus up to November 2024.	Methods 2.1
**7. Search Strategy**	Present the complete electronic search strategy.	Full search strings for each database are provided with MeSH/Emtree terms.	Methods 2.1
**8. Selection of Sources of Evidence**	Describe the process for selecting evidence sources.	Three-step screening (title, abstract, full text) conducted by two reviewers with consensus resolution.	Methods 2.2
**9. Data Charting Process**	Describe methods for charting data (who, how, and tools used).	Standardized tables were created to extract data for original and review studies.	Methods 2.4–2.5
**10. Data Items**	List and define all variables extracted.	Variables: author, year, design, population, gestational period, objectives, key findings, limitations.	Methods 2.4–2.5
**11. Critical Appraisal of Individual Sources (optional)**	If performed, describe the methods used.	No formal scoring, but study quality and strength of evidence were qualitatively assessed.	Methods 2.2 / Discussion
**12. Synthesis of Results**	Describe methods for summarizing and analyzing data.	Qualitative synthesis organized into four thematic domains of CVR in pregnancy.	Methods 2.3
**13. Results – Selection of Sources**	Report number of sources screened, assessed, and	205 records identified, 20 included; PRISMA flow diagram presented.	Figure 1 / Methods 2.2

Item	PRISMA-ScR Requirement	How It Was Addressed in the Manuscript	Section / Page
	included, with reasons for exclusion.		
**14. Characteristics of Sources**	Present characteristics for each included source.	Detailed in Tables 1–8 (design, population, findings, limitations).	Results
**15. Critical Appraisal of Sources**	Present results of any quality assessment.	Discussed narratively with emphasis on sample size, design, and generalizability.	Results / Discussion
**16. Results of Individual Sources**	Present data relevant to each research question.	Data organized in thematic tables with outcomes and limitations.	Results
**17. Synthesis of Results**	Summarize main results in relation to objectives.	Integrated synthesis presented across thematic domains.	Results / Discussion
**18. Summary of Evidence**	Summarize overall evidence and relevance.	Discussion provides comprehensive summary and clinical interpretation.	Discussion
**19. Limitations**	Discuss limitations of evidence and review process.	Addresses heterogeneity, selection bias, and limited representation of low-resource settings.	Discussion 4.2
**20. Conclusions**	Provide interpretation of findings and implications.	Conclusions summarize evidence and call for future research and clinical application.	Conclusions
**21. Funding**	Describe sources of funding and their role.	"This research received no external funding."	End of manuscript
